# Evaluation of tracheostomy suctioning procedure among nursing and respiratory therapy students: wearable manikin vs. standard manikin

**DOI:** 10.3389/fmed.2023.1220632

**Published:** 2023-12-07

**Authors:** Kevin Lumowa, Kin Long Lui, Noha Daher, Caroline Baek, Laren D. Tan, Abdullah Alismail

**Affiliations:** ^1^Department of Cardiopulmonary Sciences, School of Allied Health Professions, Loma Linda University Health, Loma Linda, CA, United States; ^2^Adventist Health White Memorial, Los Angeles, CA, United States; ^3^Allied Health Studies, School of Allied Health Professions, Loma Linda University Health, Loma Linda, CA, United States; ^4^School of Nursing, Loma Linda University Health, Loma Linda, CA, United States; ^5^Department of Pulmonary, Critical Care, Hyperbaric, Allergy and Sleep Medicine, Loma Linda University Health, Loma Linda, CA, United States; ^6^Department of Medicine, School of Medicine, Loma Linda University Health, Loma Linda, CA, United States

**Keywords:** simulation, tracheostomy, suctioning, respiratory, nursing

## Abstract

**Introduction:**

This study aims to evaluate cognitive load (CL), emotional levels (EL), and stress levels (SL) of students when using a wearable manikin vs. a standard manikin for tracheostomy suctioning (TS).

**Methods:**

This study was approved by the Institutional Review Board. Subjects were recruited by email. Subjects completed a baseline demographics questionnaire, then they were randomized into two groups: wearable manikin group (WMG) or standard manikin group (SMG). For the WMG, an actor simulated a patient by wearing the device. In phase I, both groups were educated on how to perform TS by video and offered hands-on practice. Then I put through a tracheostomy suctioning clinical simulation and completed a post sim-survey. In phase II, the same survey was repeated after encountering a real patient as part of their clinical rotation.

**Results:**

A total of 30 subjects with a mean age 26.0 ± 5.5 years participated. 20 (66.7%) were respiratory care students and 10 (33.3%) were nursing students. In the WMG, the median stress level dropped significantly post phase II compared to post phase I [2(1,4) vs.3(1,5), *p* = 0.04]. There were no significant changes in median CL, confidence, and satisfaction levels between post phase II and post phase I (*p* > 0.05). In the SMG, the satisfaction level increased significantly post phase II compared to post phase I [5(4,5) vs.4(2,5), *p* = 0.004], but there were no significant changes in CL, SL, and confidence levels between post phase I and phase II. There was no significant difference in mean EL scores over time and these changes did not differ by group. Subjects in the WMG showed a higher mean competency score than those in the SMG (85.5 ± 13.6 vs. 78.5 ± 20.8, *p* = 0.14, Cohen’s *d* = 0.4), yet not significant.

**Conclusion:**

Our results showed that the WMG is beneficial in helping bridge the gap of learning TS from the sim setting to the real-world clinical setting. More studies with higher sample size and use of other CL scales that assesses the different types of CL are needed to validate our findings.

## Introduction

Tracheostomy is a procedure where an opening in the trachea is made, exteriorizing it to the skin of the neck and producing a temporary fistula/opening. This procedure is typically done as an alternative to prolonged endotracheal intubation, providing improved patient comfort, lower airway resistance, and easier airway care (1). Tracheostomy care is arguably as important as the procedure. Complications of tracheostomies include infection, hemorrhage, pneumothorax, aspiration, and development of granulation tissue (2). Many complications can be prevented by good tracheostomy care and management (3, 4). In school, students typically learn about tracheostomy care with manikins through clinical simulations (5).

Clinical simulation allows exposure to the clinical setting in a safe environment, while providing real-world scenarios and preparing them for what they will encounter when they work (6). Including manikins in clinical simulation allows students to practice invasive procedures as part of a specific scenario. Despite the advantages of clinical simulation in healthcare education, one of the biggest disadvantages is the lack of human systems and interaction (7). Another method of teaching in medical education is the Objective Structured Clinical Examination (OSCE). Like clinical simulation, OSCE lets students practice and apply their clinical skills and knowledge for areas such as patient interaction, clinical examination, history taking, medical procedures/prescription, and interpretation of medical tests/results (8). However, unlike clinical simulation, OSCE utilizes standardized patients. The standardized patients are typically paid actors that can act out different scenarios as patients for students to practice their clinical skills. The scope of OSCE scenarios is limited to non-invasive procedures and physical exams. Even after a great deal of clinical training, students may not feel ready or feel nervous when taking care of patients in the real clinical setting (5).

The literature on both clinical simulations and OSCE is vast. Researchers have been using the following factors with learning such as cognitive load (CL), emotions (9–12), and stress (13–22). These factors are typically self-reported by the student after being involved in a clinical scenario. These factors vary among learners and can influence the transition from classroom to clinical setting (23, 24). Pawar et al. (18) reported on the use of CL and emotions in a multidisciplinary setting where they measured CL and emotions of nurses and medical staff in a clinical scenario (18). They found that CL was similar between both professions (18). In addition, stress and CL have been reported to be a critical tool to measure in simulation scenarios and sometimes it may be higher compared to real world settings (13). Negative emotions have been reported to be associated with higher CL in difficult and stressful scenarios (14).

The influence of CLT, developed by Sweller (25), in medical education tasks and procedures have been reported widely in the literature and linked to tasks and procedures by healthcare professionals (13, 16, 18, 20–22, 25–33). CLT has the following components: memory systems such as working and long term memory, learning process, and the effect of CL on memory systems (33). There are three main loads when trying to evaluate or measure CL: Intrinsic, Extraneous, and Germane loads. Researchers have studied and measured CL using different validated instruments in medical education during different types of procedures, settings, and environments. These studies typically tried to unpack and understand other factors with CL such as role of CL with learning, CL and emotions, CL and crises situations where stress is also involved (13–15, 18, 28, 31, 34).

Therefore, the purpose of this study was to evaluate stress, cognitive load, and emotion of students using a unique new technology, wearable manikin, vs. a standard manikin for tracheostomy suctioning in a simulation settings The secondary purpose of this study was to compare tracheostomy suctioning competency scores of students using a wearable manikin vs. a standard manikin.

## Methods

This study was approved by the Institutional Review Board at Loma Linda University, Loma Linda, CA, United States. This was a prospective randomized interventional study. Inclusion criteria for subjects were respiratory therapy students and nursing students. Exclusion criteria for subjects was anyone who had prior healthcare experience prior to getting to either field of study that involves tracheostomy care. For example, a nursing student who was previously a respiratory therapist or the other way around would fit the exclusion criteria. This would apply to any healthcare career that involves tracheostomy suctioning or has experience in performing suctioning before entering the current discipline. The study subjects were recruited by email via their program directors who sent the flyers and invitation to them. After reviewing and signing the informed consent with the study investigators, subjects were scheduled to meet the study investigators at the simulation center (Loma Linda University Medical Simulation Center). Subjects were then randomized into two groups using an excel randomization table sheet. The study had two phases, Phase I and Phase II.

### Outcome measures

#### PANAS scale

The emotion of subjects was measured using the validated Positive and Negative Affect Scale (PANAS) (35). The PANAS displays a very good internal reliability that is consistent with Cronbach alpha coefficient scores ranging from 0.86 to 0.90 for the Positive Affect Scale and 0.84 to 0.87 for the Negative Affect Scale (35–38). For the scoring of the PANAS scale, subjects responded to 20 different emotions, 10 positive emotions, and 10 negative emotions. For each emotion, a score was marked on a scale from “Very slightly or not at all” to “Extremely.” The positive and negative emotions scores were then added up separately to generate a score that ranged from 10 to 50. Higher scores represent higher levels of that emotion, and lower scores represent lower levels of that emotion. For example, higher scores for positive affect represent higher levels of positive emotions, and lower scores for negative affect represent lower levels of negative emotions.

#### Cognitive load

Cognitive load was assessed using a validated scale by Paas et al. where cognitive load was measured using a nine-point Likert scale that ranges from very very low mental effort to very very high mental effort. The cognitive load displays strong internal consistency with Cronbach’s α = 0.86 (34). Subjects responded to the following question: “in this tracheostomy simulation, I invested” (23, 29).

#### Stress and confidence levels

A self-reported question on stress level was used to rate the subject’s stress level using a five-point Likert scale answers ranging from (strongly agree to strongly disagree). Confidence level was assessed by asking the subjects to respond to: “I felt confident in performing tracheostomy suctioning procedure.” Responses were based on a five-point Likert scale ranging from strongly agree to strongly disagree.

#### Competency check off

A competency check off was developed by the authors where the recorded video content highlighted the same competency check off steps. See Online Supplementary Table.

## Phase I

After signing the informed consent, all subjects were asked to complete a baseline questionnaire. The questionnaire included questions about the subjects’ demographics, experience with tracheostomy care, and the PANAS scale to measure their emotions at baseline. Next, they were educated on how to perform tracheostomy suctioning through an educational video that was developed by the study investigators. Once subjects watched the educational video, they were given 15 min to orient themselves with the suctioning equipment and ask any questions regarding the suctioning procedure. Lastly, subjects went through a clinical scenario involving a tracheostomy suctioning competency according to their group assignments. Both groups were checked off using the same competency check list.

### Group 1

**Figure 1:**
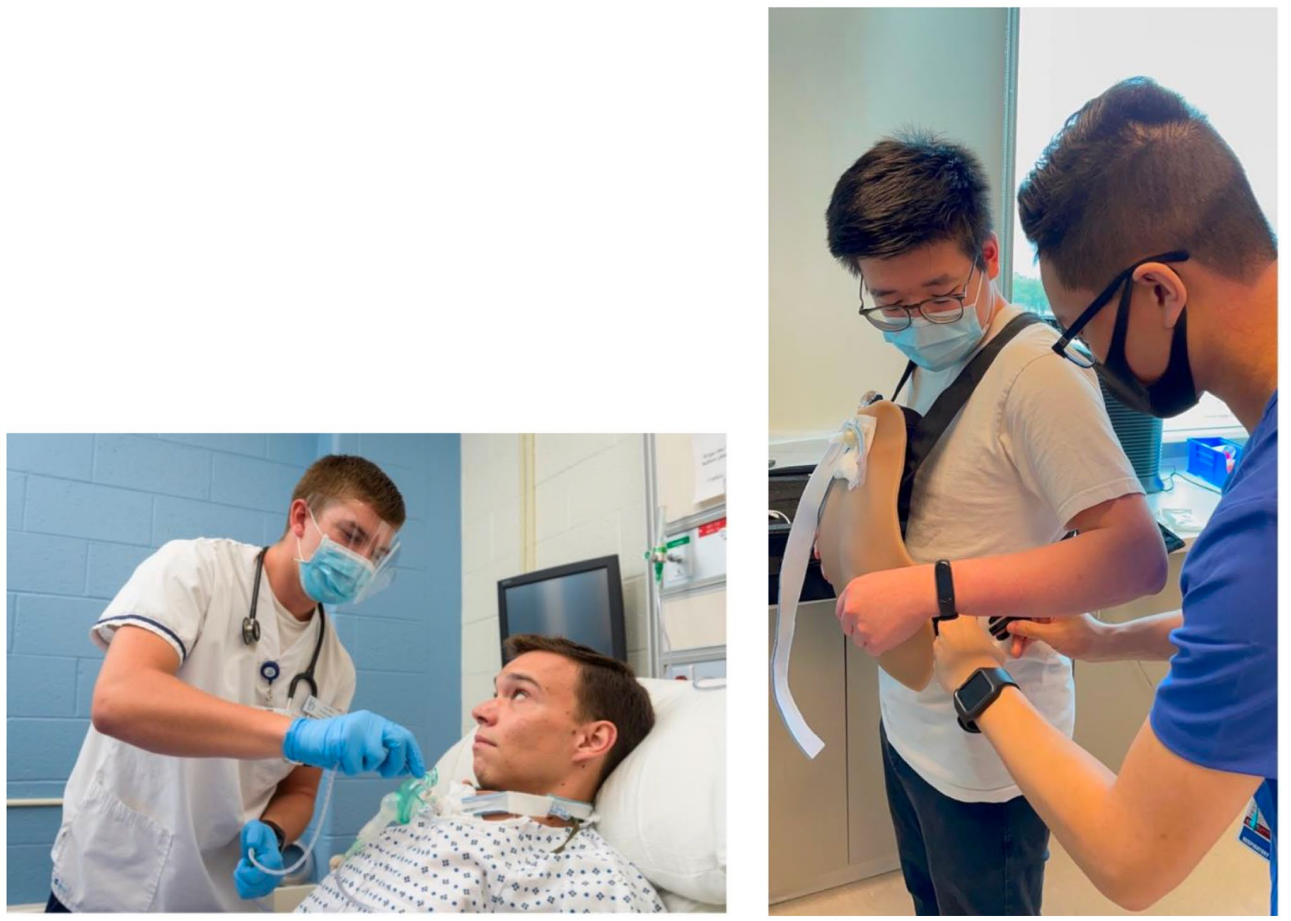
From the left, picture showing an actor wearing the Avtrach wearable manikin and a student performing tracheal suctioning. Picture was taken from Avkin.com; used with permission from Avkin.com. Right picture shows study investigators getting prepared by wearing the AvTrach manikin by Avkin.

The Wearable Manikin Group (WMG) utilized a manikin called “AvTrach” by Avkin (https://avkin.com/avtrach-product/; Figure 1). As subjects walked into the simulation room, an actor was present wearing the Avkin manikin with a hospital gown. The same actor was used for all subjects in this group. The actor and simulation technician and/or educator in the control room were connected via phone where the personnel in the control room were giving hints to the actor based on the case. The simulation technician and/or educator also controlled the breath sounds on the manikin using an application. When the subject started suctioning the patient (actor), the manikin vibrated if the subject went too far in the trach which prompted the actor to cough in response to the vibration cue.

### Group 2

Standard Manikin Group (SMG), which utilized high-fidelity adult manikin (Laerdal SimMan 3G). Subjects simply walked into the room with the manikin there along with the vital signs’ monitor and the simulation personnel in the control room. They performed the suctioning procedure using the high-fidelity manikin.

The clinical scenario for both groups was as follows:

Scenario: You are a [respiratory therapist or nurse] at a hospital called to assess a patient with a tracheostomy tube in the adult unit. The patient has recently been coughing more frequently and you are called to assess the patient accordingly.

Room presentation: ICU room, patient lying in bed in a semi-fowler position receiving oxygen via a tracheostomy mask. The patient is exhibiting an increased work of breathing, and their breath sounds shows bilateral course crackles.

Once the scenario is completed, subjects in both groups were debriefed with the study investigators about their performance as well as provided feedback. Subjects then completed the post-phase 1 survey, which included questions about the following domains: emotions using PANAS scale, cognitive load scale, and stress level.

## Phase II

**Figure 2:**
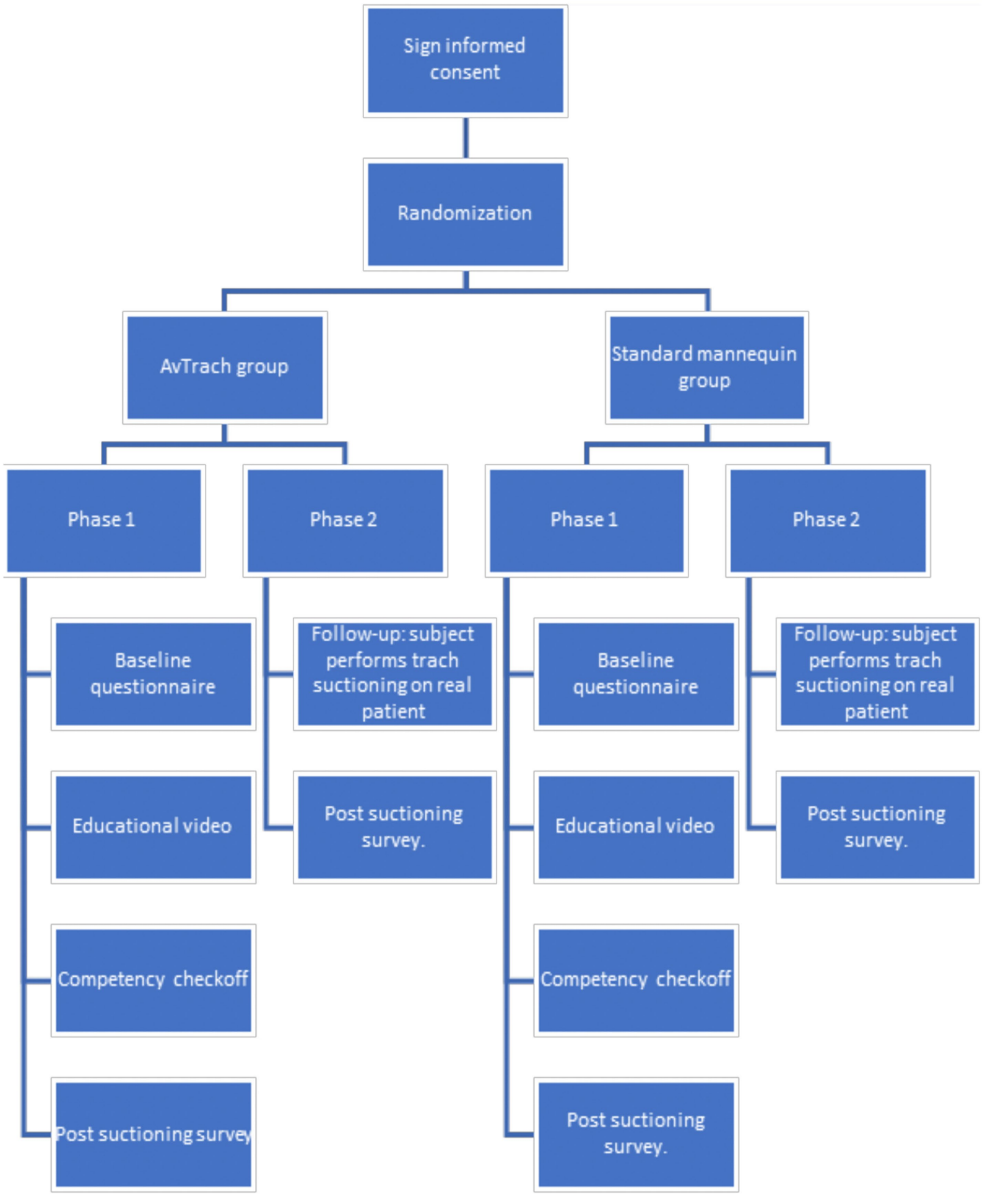
Flow diagram of the study.

For phase 2 of the study, subjects were asked to complete a questionnaire that addresses the same domains as above (PANAS, Cognitive Load, and Stress) after suctioning a real tracheostomy patient as part of their clinical rotation. Figure 2 below shows a flow diagram of the study.

### Data analyses

Statistical analyses were conducted using SPSS version 28.0. Data were summarized using frequencies and percentages for categorical variables, mean ± standard deviation (SD) for quantitative variables, and median (minimum, maximum) for ordinal variables. The normality of the quantitative outcomes was examined using Shapiro wilk test and normality plots. The subjects’ baseline characteristics were compared between the two groups using chi-square test of independence for qualitative variables, independent *t*-test for continuous variables, and Mann–Whitney U test for outcome variables that were not symmetrical or ordinal. Changes in cognitive load, stress, confidence, and satisfaction between phase I and phase II were compared using Wilcoxon signed rank test for each group separately. In terms of emotions, changes in positive and negative PANAS scores over time (baseline vs. post phase I vs. post phase II) by group (WMG vs. SMG) were examined using mixed factorial ANOVA. Mean competency for tracheostomy suctioning score was compared between the two groups using independent *t*-test. The level of significance was set at *p* < 0.05.

## Results

Thirty subjects with a mean age of 26.0 ± 5.5 years participated in this study. The majority were females (*n* = 23, 76.7%) and over half were trained in tracheostomy care (*n* = 17, 56.7%). Twenty subjects were respiratory therapists (RT) and 10 were registered nurses (RN). Sixteen subjects (46.7%) were in their first year, 11 (36.6%) in the second year, and 5 (16.7%) in the third year. The frequency distribution of subjects’ characteristics by study group is displayed in Table 1. There were no significant differences between the two groups in age, gender, profession, year in program, and number of times suctioning a manikin or a patient (*p* > 0.05, Table 1).

**Table 1:** Frequency distribution (%) of participants’ characteristics by study group (*N* = 30).

Characteristics	WMG (*n*_1_ = 15)	SMG (*n*_2_ = 15)	*p* value
Age (mean ± SD)	25.9 ± 6.6	26.2 ± 4.1	0.867
Gender			0.5
Male	4 (26.7)	3 (20.0)	
Female	11 (73.3)	12 (80.0)	
Profession			0.35
Respiratory Care	9 (60.0)	11 (73.3)	
Nursing	6 (40.0)	4 (26.7)	
Year in Program			0.75
1st year	6 (40.0)	8 (53.3)	
2nd year	6 (40.0)	5 (33.3)	
3rd year	3 (20.0)	2 (13.4)	
Trained in tracheostomy suctioning			0.5
Yes	9 (60.0)	8 (53.3)	
No	6 (40.0)	7 (46.7)	
Number of times performing tracheostomy suctioning on a manikin^*^	0 (0, 4)	0 (0, 3)	0.33
Number of times performing tracheostomy suctioning on a patient^*^	6 (0, 25)	2 (0, 20)	0.463

WMG, Wearable manikin group; SMG, Standard manikin group; SD, Standard deviation.^*^median (minimum, maximum).

Changes in median (minimum, maximum) cognitive load, stress, confidence and satisfaction levels between post phase II and post phase I are shown in Table 2. In the WMG, the median stress level dropped significantly post phase II compared to post phase I [2(1,4) vs.3(1,5), *p* = 0.04]. However, there was no significant change in median (minimum, maximum) stress between post phase II and post phase I in the SMG [2(1,4) vs.3(1,5), *p* = 0.14]. In the SMG, the median satisfaction level increased significantly post phase II compared to post phase I [5(4,5) vs.4(2,5), *p* = 0.004], but not in the WMG [2(1,5) vs. 2(1,4), *p* = 0.09]. However, there were no significant changes in median (minimum, maximum) cognitive load, and confidence between post phase II and phase I in the WMG and SMG groups (*p* > 0.05, Table 2).

**Table 2:** Median (minimum, maximum) of cognitive load, stress, confidence, and satisfaction within each study group.

	WMG (*n*_1_ = 15)		*p* value^*^	SMG (*n*_2_ = 15)		*p* value^*^
	Post phase 1	Post phase 2		Post phase 1	Post phase 2	
Cognitive Load	5 (1, 8)	5 (1, 8)	0.34	5 (1, 8)	5 (1, 8)	0.61
Stress	3 (1, 5)	2 (1, 4)	0.04	2 (1, 4)	2 (1, 5)	0.14
Confidence	4 (1, 5)	4 (1, 5)	0.34	4 (2, 5)	5 (4, 5)	0.16
Satisfaction	4 (2, 5)	4 (1, 5)	0.09	4 (2, 5)	5 (4, 5)	0.004

WMG, Wearable manikin group; SMG, Standard manikin group.^*^Wilcoxon Signed Ranks Test.

Results of the general model mixed factorial ANOVA showed that there was no significant difference in mean positive PANAS score over time (*F*_2,56_ = 1.6, *p* = 0.21, ƞ^2^ = 0.05) and these changes did not differ by group as determined by group × time effect (*F*_2,56_ = 0.6, *p* = 0.53, ƞ^2^ = 0.02). Similarly, there were no significant changes in mean negative PANAS score over time (*F*_2,56_ = 1.3, *p* = 0.27, ƞ^2^ = 0.05) and these changes did not differ by group as determined by group × time effect (*F*_2,56_ = 0.4, *p* = 0.67, ƞ ^2^ = 0.01, Table 3).

**Table 3:** Changes in mean ± SD PANAS positive and negative emotions over time and by group.

	WMG (*n*_1_ = 15)			SMG (*n*_2_ = 15)			*p* value (over time)	*p* value (group × time)
	Baseline	Post phase 1	Post phase 2	Baseline	Post phase 1	Post phase 2		
Positive	38.1 ± 6.3	40.1 ± 6.6	39.1 ± 8.8	35.9 ± 7.1	37.4 ± 5.7	39.1 ± 6.6	0.21	0.53
Negative	13.2 ± 3.5	13.6 ± 5.5	12.7 ± 2.0	13.7 ± 2.8	13.3 ± 4.4	11.7 ± 2.3	0.27	0.67

WMG, Wearable manikin; SMG, Standard manikin group; and SD, Standard deviation.

When comparing competency between the two groups, subjects in the WMG showed a higher mean competency score than those in the SMG (85.5 ± 13.6 vs. 78.5 ± 20.8, *p* = 0.14, Cohen’s *d* = 0.4), yet not significant.

## Discussion

In this study, emotion was measured at three different times, baseline, post phase I, and post phase II. Overall, there was no significant difference in mean positive PANAS affect score over time and these changes did not differ by group as determined by group × time effect. Similarly, there was no significant difference in mean negative PANAS affect score over time, and these changes did not differ by group as determined by group × time effect. Subjects in the WMG had slightly higher positive emotions at baseline and post phase I. However, at post phase II, both groups had the same level of positive emotions. Positive and negative emotional states can influence an individual’s learning differently (18). Positive emotions can encourage individuals to focus on the big picture of a learning session while negative emotions can influence an individual to focus on specific details associated with a learning scenario, which can be useful in tasks requiring an attention to detail (39–41). We speculate that this may be due to the time in between post phase 1 and post phase 2 for the subjects. Completion of phase 2 for subjects was dependent on them finding a patient with a tracheostomy tube during their clinical rotations, which is unpredictable. Some subjects completed phase II within a couple days, and some completed phase II within couple weeks. This factor of time could have affected the subjects’ emotions in post phase II.

Joels et al. (42) predicted that stress experienced within the context of a learning experience will induce focused attention and improve memory of relevant over irrelevant (later) information. Not all stress is bad, it can be beneficial for learning. In healthcare education, instructors are expected to manage the type and amount of stressors that are experienced by learners and utilize it to induce learning (43). In this study, stress was measured from post phase I to post phase II. There were no significant changes in median stress between post phase II and phase I for the SMG. However, in the WMG, the median stress level dropped significantly post phase II compared to post phase I. We believe that results can vary with high scores of stress. In addition, there are conflicting data regarding the association of stress and performance when it comes to performance (17, 44, 45).

Cognitive load, confidence, and satisfaction levels were at the same level across all groups, post phase I and post phase II. There were no significant changes in median cognitive load, confidence, and satisfaction levels between post phase II and post phase I. In the SMG, the median satisfaction level increased significantly post phase II. Measuring cognitive load has been a persistent challenge for educational researchers (46). One of the most popular ways to measure it is via self-report using a scale developed by Paas (23, 29, 33). In this study, both groups exhibited nearly the same level of cognitive load: nearly low nor high mental effort. In a study by Fraser et al., they found that cognitive load between 3 and 6 out of 9 was associated with maximal learning experience and scores above 7 resulted in declined performance (15, 47). Both groups experienced an effective level of cognitive load suitable for learning. We speculate this partly due to the baseline education not being too overwhelming and the clinical scenario being straightforward and not too extraneous. Multiple studies suggested that training conditions and learning materials, rather than pre-existing knowledge, represent the main determinant of cognitive load (15, 28).

For the secondary objective, subjects in the WMG group showed a higher mean competency score than those in the SMG. However, this difference was not significant. We speculate the reason subjects in the WMG had a higher competency was due to a more realistic experience of interacting with a patient actor compared to a manikin. Moreover, competency-based model (CBM) and cognitive load (CL) is widely discussed in medical education when it comes to creating a clear link/relationship between the two variables (CL and CBME) (33). The development and creation of entrustable professionalism activity is one of the suggested assessment and evaluation models that are used in some medical fields (primarily medicine) (33, 48). Unfortunately, the two professions that we used, nursing and respiratory therapy students, are in the early stages of using EPA as an assessment and evaluation method in their profession (49–51). Thus, based on our findings, we think that mostly likely the reasons for WMG having a higher competency score was due to having more realistic experience with the settings of the simulation, having a patient actor. In addition, in this study we assessed CL using Paas Scale where it measures overall CL and not the deeper components of CL, (intrinsic, extraneous, and germane loads) such as cognitive load components and NASA Task Load Index questionnaires. We thus think that if we have assessed extraneous load (which assesses the presentation of the task/procedure) and germane load (how the learner processes the procedure for learning purposes), we might have a better understanding of the link between CL and competency scores (30, 31). Lastly, we believe that our low sample could also be a factor and more studies that have a higher sample and deeper assessment of CL components might shed better light on understanding the connection between both concepts.

### Limitations

The sample size for this study was relatively small for generalizability. In addition, subjects from this study were recruited from one institution. Having a multi-center study might elaborate more on the findings. Moreover, the unpredictability of when students would have the ability to treat a patient with a tracheostomy tube could have influenced the results in post phase II. After a long enough time, subjects could not remember all or some of the information learned from phase I to be utilized for phase II. Therefore, adding the time factor in future studies might be beneficial to limit recall bias. Lastly, future studies should consider having another competency check off at the bedside where a study investigator (or clinical instructor) performs another check off. This way, learning can be assessed at multiple time points using manikin and real patients.

## Conclusion

Based on our findings in this study, there was not a significant change in cognitive load and stress between post phase I and post phase II. For emotion level, there was no significant change in mean positive and negative emotions over time. However, for those in the WMG, stress significantly decreased from post phase I to post phase II and they showed higher mean competency scores in phase I than those in the SMG, yet not significant. Therefore, we believe that based on the results of this study, WMG is beneficial in helping bridge the gap of learning tracheostomy suctioning from the simulation setting to the real-world clinical setting. Further studies to evaluate the true clinical implication to clinical practice are needed.

## Data availability statement

The datasets presented in this article are not readily available because of subject confidentiality. Requests to access the datasets should be directed to the corresponding author, AA: aalismail@llu.edu.

## Ethics statement

The studies involving humans were approved by Institutional Review Board at Loma Linda University Health. The studies were conducted in accordance with the local legislation and institutional requirements. The participants provided their written informed consent to participate in this study. Written informed consent was obtained from the individual(s) for the publication of any potentially identifiable images or data included in this article.

## Author contributions

KeL, KiL, and AA designed the study. KeL performed literature review, study design, data collection, analysis, and manuscript writing. KiL performed data collection and manuscript writing and review. ND performed statistical analysis and manuscript writing. CB performed data collection and manuscript writing and review. LT assisted with manuscript writing and review. AA is the principal investigator for the study, manuscript review, analysis, and writing. All authors contributed to the article and approved the submitted version.
