# Supplementary material for: Evaluation of tracheostomy suctioning procedure among nursing and respiratory therapy students: wearable manikin vs. standard manikin
Source: Front Med (Lausanne). 2023 Dec 7;10:1220632. doi: 10.3389/fmed.2023.1220632 (PMC10734638; doi:10.3389/fmed.2023.1220632)
Supplement: Supplementary file 1 [file Table_1.docx]

Tracheostomy suctioning competency

Trach suctioning competency

|  | Competent | Incompetent |
| --- | --- | --- |
| Introduce yourself to the patient |  |  |
| Confirm patient ID |  |  |
| State your reason for visit |  |  |
| Perform hand hygiene |  |  |
| Listen to breath sounds |  |  |
| Check vital signs |  |  |
| Turn on suction vacuum system |  |  |
| Apply sterile gloves using sterile technique |  |  |
| Remove patient's oxygen device with the dirty hand |  |  |
| Placing catheter into trach with the clean hand |  |  |
| Suction patient for no more than 10 sec |  |  |
| Reapply oxygen device to patient |  |  |
| Discard soiled equipment |  |  |

General comments

________________________________________________________________

________________________________________________________________

________________________________________________________________

________________________________________________________________

________________________________________________________________
